# MedEdTrials: Protocol registration for medical education research

**DOI:** 10.15694/mep.2019.000125.1

**Published:** 2019-06-07

**Authors:** Ken Masters

**Affiliations:** 1 Sultan Qaboos University

**Keywords:** publication bias, outcome switching, medical education, medical education journals, negative results, Clinicaltrials.gov, Study 329, ethics

## Abstract

This article was migrated. The article was marked as recommended.

Publication bias (the tendency for studies with positive results to be more easily published than studies with null or negative results) and outcome switching (the adjustment of study aims to match results) have long been problematic in medical research. Recent legislation, protocol-registration sites, and agreements by medical journals have led to a reduction of these problems in medical research. In medical education specifically, however, there is no such legislation, registration, or agreement. This paper argues for the creation of such a registration site and agreement by medical education journals as a matter of ethical necessity.

## Introduction

### Problems with evidence: publication bias and outcome switching

Researchers and practitioners in evidence-based fields rely upon quality evidence, and attempt to follow recommendations indicated by successful interventional and observational studies. Two problems that prevent the true picture of evidence from reaching a wider audience are *publication bias* and *outcome switching.*

*Publication bias* is the tendency of research studies with positive results to be more easily published than research studies with null or negative results. ( Sterling, 1959; Hopewell *et al.*, 2009; Joober *et al.*, 2012; Hedin *et al.*, 2016) Generally, academic journals favour studies with positive results, and researchers are also loath to publish research that has failed, especially embarrassingly so. Because of the lack of comparative unsuccessful studies on any intervention, readers and practitioners will infer an exaggerated and false level of viability of that intervention. In medical research, publication bias is wide-spread and increasing. ( Hopewell *et al.*, 2009; Hedin *et al.*, 2016; Mlinarić, Horvat and Smolčić, 2017)

*Outcome switching* occurs when researchers perform an intervention, and then receive results that do not support the original research aims, and so they ignore, or retrospectively alter, their research aims to match those results. In so doing, they obscure the negative results and exaggerate (or totally falsify) the success of their intervention. In medical research, there are many examples of outcome switching ( Goldacre *et al.*, 2016); perhaps the most notorious example is *Study 329*, dealing with the drug Paxil. ( Doshi, 2015; Le Noury *et al.*, 2015; Mulder, Rucklidge and Toop, 2016)

### The problems in medical research and practice

Because evidence-based medicine relies on published research as that evidence, and publication bias and outcome switching results in tainted evidence, the impact on patient care can be devastating. ( Whittington *et al.*, 2004; Joober *et al.*, 2012; Goldacre *et al.*, 2016)

In response to these problems, Section 801 of The Food and Drug Administration Amendments Act of 2007, known as FDAAA 801, ( FDA, 2007) established the legal necessity for protocols of all clinical trials in the United States to be registered before implementation. This would mean that the full scope, goals, aims and methods of the research would be available publicly on a website beforehand, and any published results stemming from the trial could be compared to the original protocol. In addition, if no results were published, this could be questioned.

Even though this law is applicable to the United States only, its strength is reinforced by the practice of medical journals’ requiring authors to have previously registered their trials; manuscripts that do not cite a protocol registration number are rejected even before peer-review.

Although researchers can register their trials on many such sites, one of the largest sites on which these trials are registered is ClinicalTrials.gov ( https://clinicaltrials.gov/) on which researchers register clinical trials (or interventional studies) and observational studies. ( ClinicalTrials.gov, 2018)

This solution has not entirely solved the problems of publication bias and outcome switching (as industry-sponsored journals do not always follow suit), but it has been a successful step in the right direction, and this success will increase as more and more medical journals adopt the practice.

## Medical Education

Although publication bias and outcome switching do not appear to be well-studied in medical education, publication bias has been noted in social sciences for more than 60 years ( Sterling, 1959; Fanco, Malhorta and Simonvits, 2014), and there is no reason to believe that it does not exist in medical education. In spite of this, no such protocol registration sites exist for medical education research, and medical education journals do not generally require prior registration of studies.

One might question the need for such a medical education site, on the grounds that few medical education studies are trials, and that the stakes for medical education are not as high as they are for medical research. I hold, however, that these arguments are weak:

• Similar protocol registries already exist for several social sciences, including general education. ( Anderson, Spybrook and Maynard, 2019) Medical education’s absence is distinctly, and embarrassingly, noteworthy.
• Although the name *MedicalTrials.gov* implies randomised, controlled trials, the site accepts registration of any interventional and observational study. Many medical education studies involve new interventions or implementations of interventions under different circumstances; if medical education practitioners read only about successful interventions and are not aware of the many failures, the result will be further failed attempts to implement medical education interventions that were doomed from the start.
• In medical research, there is no doubt that the stakes are high: patients’ well-being and their lives. But it is not true to say that, in medical education research, the stakes are not as high. They are identical; they are just not as obvious. The chain is longer, but attempting medical education interventions that were already doomed to failure is poor teaching, will lead to poorly-prepared medical students, and, in turn, will lead to poorly-trained health professionals; ultimately, there will be a negative impact on patients’ well-being and their lives. The chain is longer, but it is no less sure.
• Such a registry is in keeping with current publication trends in all fields towards far greater openness and transparency.

Finally, it is with some embarrassment that we note that, although medical journal editors and publishers had known about publication bias and outcome switching for many years, it was only after legal pressure in the form of FDAAA 801 that these journals required pre-registration. It would be a sad day for medical education if, in spite of the obvious existence and impact of these on medical education and healthcare, medical education journals were to wait for the lawyers to instruct them on what is, essentially, an ethical decision.

## MedEdTrials

In response to this need, the following is proposed:

• The creation of a website, *MedEdTrials.org*, in which all medical education researchers can pre-register any medical education interventional study. (For convenience, I refer to “medical”, but this site should be applicable to all human and animal health sciences education). Although the details of the registration process and requirements for each intervention registration will need to be established by all stake-holders, especially medical education journal editors (see next point), the site’s characteristics should be:
  • Free registration of researchers and protocols.
  • Researchers are identified by unique, publicly verifiable identifiers such as ORCID ( https://orcid.org/) numbers.
  • Interventions to be allocated a unique identifier.
  • When fields have been completed in the initial registration, they cannot be edited, although extra notes and documents can be added for clarification.
  • Annual updates on the progress of the research until publication, or a decision to not publish. If the decision is made to not publish, reasons must be given.
  • All entries to be date- and time-stamped.
  • Free and open access of the information to the public, without registration requirements.
• Agreement by editors of all medical education journals (and any journals publishing medical education manuscripts) that any interventional- and observational-study manuscripts submitted for publication should indicate the unique identifier issued by MedEdTrials.org (or any other similar website), and should provide a link to the protocol. Where authors have deviated from the protocol, this should be suitably justified, and part of the editorial or reviewing process should include a comparison of the protocol with the manuscript. Again, this includes all human and animal health sciences education publications.

## Conclusion

Publication bias and outcome switching have long been recognised as having a harmful impact on medical research and healthcare. These problems have been partially addressed by legislation requiring pre-registration of medical interventional and observational studies’ protocols, and agreements by medical journal editors to publish only manuscripts that indicate such registration.

This paper calls for a similar registration site and agreement for medical education. Medical educators should not wait for legislation, but should embark on this venture because it is an ethical necessity required to enhance medical education scholarship, and will impact positively on all aspects of healthcare education, and, ultimately, healthcare delivery.

## Take Home Messages

• Publication bias and outcome switching have long been problematic in medical research.
• Recent legislation, protocol-registration sites, and agreements by medical journals have led to a reduction of these problems in medical research.
• In medical education, there is no such legislation, registration, or agreement.
• This paper argues for the creation of such a registration site and agreement by medical education journals as a matter of ethical necessity.

## Notes On Contributors

Ken Masters, PhD, FDE is Associate Professor of Medical Informatics at Sultan Qaboos University, Sultanate of Oman. He has been involved in education and medical education research for some 20 years. ORCID: https://orcid.org/0000-0003-3425-5020

## Declarations

I am Associate Editor of MedEdPublish. This paper has been processed through the normal channels.

## References

[ref1] Anderson D. Spybrook J. and Maynard R. (2019) REES: A registry of efficacy and effectiveness studies in education. Educational Researcher. 48(1), pp.45–50. 10.3102/0013189X18810513

[ref2] ClinicalTrials.gov(2018) Why Should I Register and Submit Results? Available at: https://clinicaltrials.gov/ct2/manage-recs/background( Accessed: 20 May 2019).

[ref3] Doshi P. (2015) No correction, no retraction, no apology, no comment: Paroxetine trial reanalysis raises questions about institutional responsibility. BMJ. (Online),351(September), p.h4629. 10.1136/bmj.h4629 26377109

[ref4] Franco A. Malhorta N. and Simonvits G. (2014) Publication bias in the social sciences: Unlocking the file drawer. Science. 345(6203), pp.1502–1506. 10.1126/science.1255484 25170047

[ref5] FDA(2007) Food and Drug Administration Amendments Act of 2007, Public Law 110-85. USA. Available at: http://www.gpo.gov/fdsys/pkg/PLAW-110publ85/pdf/PLAW-110publ85.pdf( Accessed: 13 May 2019).

[ref6] Goldacre B. Drysdale H. Powell-Smith A. Dale A. (2016) The COMPare Trials Project. Available at: http://compare-trials.org/( Accessed: 21 May 2019).

[ref7] Hedin R. J. Umberham B. A. Detweiler B. N. Kollmorgen L. (2016) Publication Bias and Nonreporting Found in Majority of Systematic Reviews and Meta-analyses in Anesthesiology Journals. Anesthesia and Analgesia,123(4), pp.1018–1025. 10.1213/ANE.0000000000001452 27537925

[ref8] Hopewell S. Loudon K. Mj C. Ad O. (2009) Publication bias in clinical trials due to statistical significance or direction of trial results (Review). Cochrane Database of Systematic Reviews.(1), p.MR000006. 10.1002/14651858.MR000006.pub3 19160345 PMC8276556

[ref9] Joober R. Schmitz N. Annable L. and Boksa P. (2012) Publication bias: What are the challenges and can they be overcome? Journal of Psychiatry and Neuroscience. 37(3), pp.149–152. 10.1503/jpn.120065 22515987 PMC3341407

[ref10] Mlinarić A. Horvat M. and Smolčić V. Š. (2017) Dealing with the positive publication bias: Why you should really publish your negative results. Biochemia Medica. 27(3), p.030201. 10.11613/BM.2017.030201 29180912 PMC5696751

[ref11] Mulder R. Rucklidge J. J. and Toop L. (2016) Restoring Study 329: Paroxetine neither effective nor safe for adolescents. Australian and New Zealand Journal of Psychiatry,50(9), pp.922–923. 10.1177/0004867416657412 27389067

[ref12] Le Noury J. Nardo J. M. Healy D. Jureidini J. (2015) Restoring Study 329: Eficacy and harms of paroxetine and imipramine in treatment of major depression in adolescence. BMJ. (Online),351, p.h4320. 10.1136/bmj.h4320 26376805 PMC4572084

[ref13] Sterling T. D. (1959) Publication decisions and their possible effects on inferences drawn from tests of significance - or vice versa. Journal of the American Statistical Association. 54(285), pp.30–34. 10.2307/2282137

[ref14] Whittington C. J. Kendall T. Fonagy P. Cottrell D. (2004) Selective serotonin reuptake inhibitors in childhood depression: systematic review of published versus unpublished data. Lancet. 363(9418), pp.1341–1345. 10.1016/S0140-6736(04)16043-1 15110490

