# Supplementary material for: MedEdTrials: Protocol registration for medical education research
Source: MedEdPublish (2016). 2019 Aug 5;8:125. Originally published 2019 Jun 7. [Version 2] doi: 10.15694/mep.2019.000125.2 (PMC10911870; doi:10.15694/mep.2019.000125.2)
Supplement: Peer review history

# Reviewer report: Reviewer response for version 2

**DOI:** 10.21956/mep.20250.r31414

**Published:** 2019-08-07

**Authors:** Dave Wilson

**Affiliations:** 1 Cardiff University School of Medicine

This review has been migrated. The reviewer awarded 2 stars out of 5 An interesting idea, but fails to convince in terms of medical education. As the author points out neither publication bias or outcome switching has been investigated in terms of medical education. For this reason, the establishment of a registry at this stage would seem to be premature. Given the ability to provide online supplemental information for readerships of most education journals, would it be simpler to ask for the authors to upload the Research Ethics approval form?

Reviewer Expertise:

NA

No decision status is available

# Reviewer report: Reviewer response for version 2

**DOI:** 10.21956/mep.20250.r31417

**Published:** 2019-08-06

**Authors:** BALAJI ARUMUGAM

**Affiliations:** 1 TAGORE MEDICAL COLLEGE AND HOSPITAL

This review has been migrated. The reviewer awarded 5 stars out of 5 Thanks for the invite once again to review this article. Good wok by the authors and now the registration process for MedEd trials is clear and easy user friendly which will enhance the researchers to register. I hope the publication bias will definitely go down considerably if its ethically followed and properly registered under this weblink.Congrats and Thanks

Reviewer Expertise:

NA

No decision status is available

# Reviewer report: Reviewer response for version 2

**DOI:** 10.21956/mep.20250.r31416

**Published:** 2019-08-06

**Authors:** Felix Silwimba

**Affiliations:** 1 University of Lusaka

This review has been migrated. The reviewer awarded 5 stars out of 5 this paper has provided me with a better understanding of research governance. it is educative and a commendable piece of work.

Reviewer Expertise:

NA

No decision status is available

# Reviewer report: Reviewer response for version 2

**DOI:** 10.21956/mep.20250.r31415

**Published:** 2019-08-06

**Authors:** Tan Nguyen

**Affiliations:** 1 Deakin University

This review has been migrated. The reviewer awarded 5 stars out of 5 I found this paper highly commendable. There certaintly has been maturity in the medical education research space. As noted, there are limitations to academic work that often does not require ethics approval. Furthermore, protocols, whilst offers transparency, may require amendments during the research being undertaken for various administrative or practical reasons. However, there is value in such an approach. It also enables the reduction in duplicating work that may be performed elsewhere and can foster international collaboration. We should also be mindful to ensure academics are not burdened by the process.

Reviewer Expertise:

NA

No decision status is available

# Reviewer report: Reviewer response for version 1

**DOI:** 10.21956/mep.18996.r27243

**Published:** 2019-07-06

**Authors:** Hossam Hamdy

**Affiliations:** 1 Gulf Medical University

This review has been migrated. The reviewer awarded 4 stars out of 5 This article discusses an important aspect related to publication bias in medical education. The rational and argument to establish a site for registering research protocols in medical education is well justified.The literature is up to date and useful for anyone who would like to further investigate this problem.

Reviewer Expertise:

NA

No decision status is available

# Reviewer report: Reviewer response for version 1

**DOI:** 10.21956/mep.18996.r27244

**Published:** 2019-06-18

**Authors:** Gary D. Rogers

**Affiliations:** 1 Griffith University

This review has been migrated. The reviewer awarded 3 stars out of 5 This is an interesting opinion piece which expresses concern about ‘publication bias’ and ‘outcome switching’ in health professional education research. It advocates for preregistration of studies through a common site and non-publication of papers reporting studies that have not been registered in advance. The issue of publication bias is particularly important in health professional education because educators often learn at least as much from the study of innovations that haven’t performed as hoped as from those that have. The problem here though lies with journal editors (especially those of the most important journals, which now have very high rejection rates) and I am not sure that such a registry will really have much impact on that. With regard to concerns about ‘outcome switching’ however, and indeed the general thrust of the paper, I am concerned that the author appears to have taken a wholly positivist stance and, while pointing to trial registries in social science, doesn’t seem to recognise the important role of qualitative methodologies in health professional education studies. Often the process of developing and evaluating practices in our field has more of the character of action research in that we learn more about the complexities of problems as we seek to solve them. For a positivist, this might be seen as ‘outcome switching’ but for those of us who utilise the full range of investigative approaches, it is actually the development of a deeper understanding of the intricacies of the inherently complex process of learning as it applies to this situation. In summary, the proposal might have some merit for the evaluation of very precisely planned ‘interventions’, where the complex is deliberately reduced to the simple in order to ‘trial’ a particular learning method. If our journals chose only to publish those kinds of studies into the future, however, our field would be much the poorer for the change.

Reviewer Expertise:

NA

No decision status is available

# Reviewer report: Reviewer response for version 1

**DOI:** 10.21956/mep.18996.r27242

**Published:** 2019-06-09

**Authors:** BALAJI ARUMUGAM

**Affiliations:** 1 TAGORE MEDICAL COLLEGE AND HOSPITAL

This review has been migrated. The reviewer awarded 5 stars out of 5 1. Two problems that prevent the true picture of evidence like publication bias and outcome switching are discussed in this article and I personally as a researcher with ORCID ID and other research gate registered person, often come across the outcome switching events more than the publication bias. This outcome switching is problems start from the thesis work of the many doctors those who perform a research for completion of their Master degree. 2. And this paper argues for the creation of such a registration site and agreement by medical education journals as a matter of ethical necessity. 3. It’s a good move and the proposal from the author of this articlePlease clarify4. How to register and whom to register?5. Can the researchers individually also can register themselves before proceeding for the medical education researches?

Reviewer Expertise:

NA

No decision status is available

# Reviewer report: Reviewer response for version 1

**DOI:** 10.21956/mep.18996.r27241

**Published:** 2019-06-07

**Authors:** Felix Silwimba

**Affiliations:** 1 University of Lusaka

This review has been migrated. The reviewer awarded 5 stars out of 5 this is a timely editorial observation of medical education research publication. I agree with the need for a registry of medical education protocols. its informative and at the same time educative to the educators themselves.

Reviewer Expertise:

NA

No decision status is available
